# Pacemaker patients' perception of unsafe activities: a survey

**DOI:** 10.1186/1471-2261-8-31

**Published:** 2008-11-14

**Authors:** Masooma Aqeel, Azam Shafquat, Nawal Salahuddin

**Affiliations:** 1 Department of Medicine, The Aga Khan University Hospital, Stadium Road, Karachi, Pakistan; 2 Section of Cardiac Electrophysiology, National Institute of Cardiovascular Diseases, Rafiqui Shaheed Road, Karachi, Pakistan

## Abstract

**Background:**

Cardiac pacing is a recognized and widely used treatment for patients presenting with bradycardia. Physicians expect patients to return to normal activities almost immediately post implantation. However, patients themselves may perceive interference to pacemaker function by various routine activities and devices, and hence continue to lead restricted, disabled lives. The aim of this study is to determine if routine activities are perceived by pacemaker patients to interfere with their device function.

**Methods:**

A descriptive cross sectional survey was carried out on consecutive patients at the pacemaker clinic at a public hospital in Karachi, Pakistan. A 47-question tool was developed and tested. Patients' perceptions of safety of performing various routine activities, along with socio-demographic data were recorded.

**Results:**

The final sample included 93 adult patients (45% males). 41% were illiterate. 77.4% recalled receiving counselling at implantation, predominantly from the implanting physician and house staff. A considerable proportion of patients considered many routine activities unsafe including driving automobiles (28%), passing through metal detectors (31%), bending over (37%), and sleeping on the side of the pacemaker (30%). Also considered unsafe were operation of household appliances- TV/VCR (television/video cassette recorders) (53%), irons (55%)) and electrical wall switches (56%).

For nearly all variables neither literacy nor history of counselling improved incorrect perceptions.

**Conclusion:**

This study shows that our pacemaker patients perceive many routine activities as unsafe, potentially leading to disabling life style modifications. The tremendous investment in pacemaker technology to improve patient performance is not going to pay dividends if patients continue to remain disabled due to incorrect perceptions. Further studies are required to determine the reasons for these misperceptions, and to determine if these problems also exist in, and hinder, other patient populations.

## Background

Cardiac pacing today is an accepted and common treatment for patients with bradycardia, with almost 600,000 pacemakers being implanted worldwide annually [1]. In Pakistan, pacemaker distributors estimate approximately 1200 new devices being implanted each year (personal communication; Medtronic distributors for Pakistan). In addition to being a life saving procedure, pacemaker implantation helps improve patient quality of life. Cardiologists expect that most patients will be able to return to their pre-morbid level of functioning post implantation.

The sparse literature on pacemaker patients in Pakistan [2,3] did not address post implant lifestyle issues. Similarly, international studies on the topic of patients living with pacemakers have focused on the patient's sense of well being after implantation more than the patient's perception of daily routine activities with an implanted device [4,5].

During interaction with our patients, we noted that some of them were self-imposing unnecessary restrictions on routine activities that they perceived to be detrimental to the function of their device. We carried out this study to find out what activities patients with devices felt were unsafe and hence could restrict their function.

## Methods

A descriptive cross-sectional survey was conducted at the outpatient pacemaker clinic of the National Institute of Cardiovascular Diseases, Karachi (a tertiary care, public-sector cardiac specialty hospital). The study was conducted in March 2007. Consenting consecutive adult (above 18 years) patients with permanent pacemakers were included. Patients who could not speak Urdu or English were excluded. Ethical approval was obtained from the hospital's ethical committee.

A questionnaire was developed on activities routinely encountered using educational material available for pacemaker patients [6-8] and was pilot tested on 10 patients with implanted devices. These 10 patients were not included in the final sample. Modifications were made according to feedback. The final 47-question instrument (Questionnaire available on request) was administered and filled by a single interviewer (non-care provider). Illiteracy was defined as inability to read or write as reported by the respondent.

Continuous data is reported as means with standard deviation or median. Categorical data is reported as proportions. Effect of literacy, counselling at time of implantation, income level and duration since implantation of pacemaker on correct practices of daily living was assessed by the chi-squared test (Fisher's exact test was used where less than 5 were present in a category). A two-tailed p-value < 0.05 was statistically significant. The SPSS (Statistical Program for Social Sciences) software version 13.0 was used to analyze data.

## Results

120 patients were assessed of which the final sample of 93 patients (55% females) met the study criterion and who all consented to participate. Mean age was 58.6 years (+/- 13.1 years). 41% were illiterate (unable to read or write). Median reported household income per month was Pakistani Rs.10, 000 [~US $167/month], (Range Rs.500 to Rs.80, 000) (Mean Pakistan monthly household income is slightly higher Rs. 12300, US $205) [9]. 34 (37%) patients had their pacemaker implantation funded by charity. Mean duration since first implantation with pacemaker was 4.1 years (ranging from less than 1 to 15 years).

Over a quarter of the patients, 28 (30.1%) felt that they could not sleep on the side of their pacemaker. Approximately one third, 34 (36.6%), of the patients felt that they cannot bend forward (e.g. during prayers). Similar incorrect perceptions were present about several other routine daily activities including driving, climbing stairs and using the arm on the implantation side (see Table 1).

**Table 1:** Patients' perceptions of permissible activities after pacemaker implantation

**Question Asked**	**Patient Response**		
	
	**Yes** **N (%)**	**No** **N (%)**	**Don't know** **N (%)**
Can patients travel in an automobile?	85 (91.4)	8 (8.6)	-
Can patients drive an automobile?	59 (63.4)	26 (28)	8 (8.6)
Can patients bathe and swim?	92 (98.9)	1 (1.1)	-
Can patients climb stairways?	67 (72)	26 (28)	-
Can patients bend over as in prayers?	58 (62.4)	34 (36.6)	1 (1.1)
Can patients pass through metal detectors?	19 (20.4)	29 (31.2)	45 (48.4)
Can patients sleep on the side of the pacemaker?	65 (69.9)	28 (30.1)	-
Can patients move the arm on the side of their pacemaker?	75 (80.6)	18 (19.4)	-

Regarding electrical appliance usage, more than half, 52 (55.9%), of the patients felt it was unsafe to use electrical wall switches and a similar number felt it unsafe to operate different household appliances including televisions, electrical irons and sewing machines (see Table 2).

**Table 2:** Patients' perception of safety of usage of electric appliances

**Question Asked**	**Patient Response**		
	
	**Yes** **N (%)**	**No** **N (%)**	**Don't know** **N (%)**
Is it safe to use mobile telephones?	42 (45.2)	27 (29)	24 (25.8)
Is it safe to use standard (wired) telephones?	71 (76.3)	10 (10.8)	12 (12.9)
Is it safe to use electrical irons?	39 (41.9)	51 (54.8)	3 (3.2)
Is it safe to touch electrical switches?	40 (43)	52 (55.9)	1 (1.1)
Is it safe to use microwave ovens?	18 (19.4)	37 (39.8)	38 (40.8)
Is it safe to touch TV/VCR/radios?	42 (45.2)	49 (52.7)	2 (2.2)
Is it safe to operate via remote controls?	62 (66.7)	24 (25.8)	7 (7.5)
Is it safe to use electrical sewing machines?	34 (36.6)	46 (49.5)	13 (14)
Is it safe to go near/use gas ovens?	71 (76.3)	17 (18.3)	5 (5.4)

72 (77.4%) patients reported receiving lifestyle counselling at the time of pacemaker implantation; advice was given by the implanting physician in 33 (35.5%), house-staff in 34 (36.6%), nurses in 1 (1.1%), pacemaker technicians in 3 (3.2%), information booklet in 15 (16.1%) and other sources (family physician, friends, internet etc) in 7 (7.5%) cases.

Table 3 shows the percentages of patients according to their perceptions as to medical diagnostic procedures that could be considered safe by pacemaker patients

**Table 3:** Medical procedures considered safe by patients

**Procedure**	**Patient Response**		
	
	**Yes** **N (%)**	**No** **N (%)**	**Don't know** **N (%)**
X- ray	65 (69.9)	7(7.5)	21 (22.6)
Ultrasound	48 (51.6)	5(5.4)	40 (43.0)
CT Scan	7 (7.5)	8(8.6)	78 (83.9)
MR scanning	3 (3.2)	10 (10.8)	80 (86.0)

Literate and illiterate (unable to read or write) patients were compared for response to each question but there was no significant difference in their perceptions except in use of remote controls, where larger proportion of literate patients were correctly aware that it was safe to use (see Table 4). Similarly, other than in use of MR scanning there was no significant difference in perceptions of patients who had received counselling versus those who had not (see Table 5). Perceptions were also compared with level of income and duration since implantation but these too did not seem to influence patient beliefs.

**Table 4:** Influence of Literacy on Patient Perception

**Question Asked**	**No. of patients who responded correctly**		**p-value**
		
	**Literate (n = 55)**	**Illiterate (n = 38)**	
Can patients travel in an automobile?	51	34	0.582
Can patients drive an automobile?	34	25	0.634
Can patients bend over (i.e. prayers)?	34	24	0.705
Can patients pass through metal detectors?	12	7	0.791
Is it safe for patients to use mobile phones?	22	20	0.171
Is it safe for patients to touch electrical switches?	27	13	0.201
Is it safe for patients to touch TV/VCR/Radios?	29	13	0.069
Is it safe for patients to operate via remote controls?	41	21	0.026
Is it safe for patients to use electrical sewing machines?	25	9	0.059

**Table 5:** Influence of post-implantation counseling on Patient Perception

**Question Asked**	**No. of patients who responded correctly**		**p-value**
		
	**Counseled** **(N = 72)**	**Not Counselled** **(N = 21)**	
Can patients travel in an automobile?	64	21	0.11
Can patients drive an automobile?	44	15	0.271
Can patients bend over (i.e. prayers)?	44	14	0.799
Can patients pass through metal detectors?	15	4	0.917
Is it safe for patients to use mobile phones?	35	7	0.430
Is it safe for patients to touch electrical switches?	28	12	0.304
Is it safe for patients to touch TV/VCR/Radios?	30	12	0.378
Is it safe for patients to operate via remote controls?	45	17	0.187
Is it safe for patients to use electrical sewing machines?	25	9	0.765
Is it safe for patients to undergo MR scanning?	10	0	0.043

## Discussion

Physicians who implant pacemakers may feel that they have restored the patient back to their pre-morbid level of activity. However, lack of information or misinformation in patients may result in self-imposed restrictions that can adversely affect ordinary activities.

While there is considerable information about clinical indications for pacemaker implantations in patient populations similar to ours [2,3], there is little available literature on what pacemaker patients perceive as permissible activities in daily life in both developing and developed countries.

Some of these issues were addressed in a small study conducted in Sweden on a sample of 13 pacemaker patients [4]. The study demonstrated that patients did restrict their daily activities due to insufficient knowledge, avoided electromagnetic fields, and were hesitant at using mobile phones or microwave ovens. However, data in this study was interview-based with narrative reporting and cannot be used for comparison.

A study from South Africa [5] looked at 94 patients and their perceptions of living with their devices. Although this study did not look at specific daily activities, it did show that up to 50% of the patients felt handicapped after the device implantation and 53% of the patients felt they were less active after the device than before. The study did not seek specific reasons from the patients for these negative findings.

A considerable proportion of our respondents felt that pacemaker patients should not perform many routine activities including driving automobiles, climbing stairs, bending over during prayers or sleeping on the side of the pacemaker. All these activities are generally considered safe for pacemaker patients to perform [6-8]. Although it has been shown that pacemaker patients can pass safely through metal detectors [7,10], a majority of our patients did not know so.

Although mobile phones have been shown to be safe in patients with pacemakers [11], provided they are kept 6 inches (15 cm) away from the device [6,8], only 45% of our patients knew that they could use their mobile phones safely.

Almost half of the patients felt that it was unsafe to touch electrical wall switches, electrical iron or operate devices like televisions or radios. This would be expected to severely restrict pacemaker patients ability to function independently even though all of these appliances are safe to use with modern pacemakers [6,8].

Most of our patients did not know that they should avoid an MRI procedure, which is currently contraindicated in pacemaker patients [12,13].

Other than one variable, there were no significant differences between the perceptions of illiterate and literate patients. This may be because the study was not powered to detect these differences or it may be an indication that education is not the only factor influencing patient behaviour in a complex socio-cultural environment.

Similarly counselling of patients did not seem to have made much impact other than in knowledge of MR scanning. One reason for this may be that our study was limited in that we could not assess the level or quality of counselling provided at time of implantation. Other reasons that counselling did not appear to alter perceptions may be that our sample size was small, there was lack of standardized counselling, and prevalent socio-cultural myths maybe stronger influences than education by health care providers.

As our study was not designed to look at the causes of misperceptions in our patients, we can only conjecture on some possible contributors. These would include lack of a system to formally educate all patients at time of implantation, lack of own knowledge of health care providers, lack of available information in multiple local languages, local myths surrounding implanted electrical devices and poor literacy rates precluding patients from self educating.

Educated societies have a large amount of information available for pacemaker patients from multiple sources i.e. brochures [6-8], support groups, and the internet. It is difficult to predict the benefit patients have from this information, as we were unable to find literature on the impact of these interventions. Nevertheless, this study does show that more effort is needed to improve the knowledge of patient cohorts similar to ours. Based on the demographics of our study, pacemaker educational material needs to be developed for patients with limited literacy and resources such as ours.

One of the major limitations of our study is that we only looked at misperceptions that are prevalent in our patients but did not investigate the extent to which these mistaken beliefs actually affected quality of life. Further studies to assess relationships between mistaken beliefs and quality of life might better quantify the actual burden caused by these misperceptions. Similarly studies need to be conducted in settings similar to ours after instituting educational interventions to see if they improve patient attitudes. Moreover, it would be interesting to see if patients in societies with more resources and education are truly better informed and lead less restricted lives.

## Conclusion

This study highlights the handicap that patients with pacemakers continue to face despite having received a 'curative procedure'. Our patients perceive many safe and permissible activities as being unsafe, and not permitted. These misperceptions were present in all facets of life whether it be routine activities at home or employment and even in the physical aspects of praying. Further work to find possible reasons for these misconceptions needs to be done and solutions based on the demographics of the patients need to be sought. Our patients need to be educated using materials for a population with limited literacy and resources. Little information is available for other populations and studies similar to this may unveil hidden misconceptions even in more educated societies.

## Competing interests

The authors declare that they have no competing interests.

## Authors' contributions

AS conceived of the study. MA collected, analyzed, and interpreted the data. AS, MA and NS wrote the manuscript. All authors have read and approved the final manuscript.

## Pre-publication history

The pre-publication history for this paper can be accessed here:


